# Development of MDS in Pediatric Patients with GATA2 Deficiency: Increased Histone Trimethylation and Deregulated Apoptosis as Potential Drivers of Transformation

**DOI:** 10.3390/cancers15235594

**Published:** 2023-11-26

**Authors:** Franziska Schreiber, Guido Piontek, Yuki Schneider-Kimoto, Stephan Schwarz-Furlan, Rita De Vito, Franco Locatelli, Carole Gengler, Ayami Yoshimi, Andreas Jung, Frederick Klauschen, Charlotte M. Niemeyer, Miriam Erlacher, Martina Rudelius

**Affiliations:** 1 Institute of Pathology, Ludwig-Maximilians-University Munich, 80337 Munich, Germany; franziska.schreiber@med.uni-muenchen.de (F.S.); guido.piontek@med.uni-muenchen.de (G.P.); yuki.schneiderkimoto@med.uni-muenchen.de (Y.S.-K.); andreas.jung@med.uni-muenchen.de (A.J.); frederick.klauschen@med.uni-muenchen.de (F.K.); 2 Institute of Pathology, Klinikum Kaufbeuren-Ravensburg, 87600 Kaufbeuren, Germany; stephan.schwarz@pathologie-memmingen.de; 3 Institute of Pathology, University Hospital Erlangen, 91054 Erlangen, Germany; 4 Department of Pathology, Bambino Gesu Children’s Hospital, IRCCS, 00165 Rome, Italy; rita.devito@opbg.net; 5 Department of Pediatric Hematology and Oncology, Cell and Gene Therapy, Bambino Gesu Children’s Hospital, IRCCS, 00165 Rome, Italy; franco.locatelli@opbg.net; 6 Department of Life Sciences and Public Health, Catholic University of the Sacred Heart, 00168 Rome, Italy; 7 Institute of Pathology, Department of Laboratory Medicine and Pathology, Lausanne University Hospital, Lausanne University, CH-1011 Lausanne, Switzerland; carole.gengler@chuv.ch; 8 Department of Pediatrics and Adolescent Medicine, Division of Pediatric Hematology and Oncology, Medical Center, Faculty of Medicine, University of Freiburg, 79110 Freiburg, Germany; ayami.yoshimi@uniklinik-freiburg.de (A.Y.); charlotte.niemeyer@uniklinik-freiburg.de (C.M.N.); miriam.erlacher@uniklinik-freiburg.de (M.E.); 9 German Cancer Consortium (DKTK), Partner Site Munich, and German Cancer Research Center (DKFZ), 69120 Heidelberg, Germany; 10 German Cancer Consortium (DKTK), Heidelberg and Freiburg, 79106 Freiburg, Germany

**Keywords:** myelodysplastic syndrome, GATA2 deficiency, apoptosis, childhood, pediatric

## Abstract

**Simple Summary:**

GATA2 deficiency is a complex disorder associated with an increased risk for myelodysplastic syndrome (MDS) and acute myeloid leukemia. In this study, we focused on pediatric MDS patients with or without an additional GATA2 deficiency and investigated the possible mechanisms underlying disease progression. We found that disease progression was associated with an upregulation in *GATA2* mRNA levels, along with the reactivation of *EZH2*, a gene controlled by *GATA2*. This was accompanied by increased histone trimethylation, a key epigenetic mark linked to *EZH2* function. Additionally, we found elevated levels of the antiapoptotic protein BCL2 in patients with an advanced GATA2 deficiency, together with alterations in apoptosis-related proteins. These findings suggest the potential drivers of disease progression in pediatric GATA2 deficiency, including increased histone trimethylation and deregulated apoptosis. Therefore, this study provides a rationale for the use of the therapeutic agents venetoclax and azacitidine, offering promising options for improving patient management in the future.

**Abstract:**

GATA2 deficiency is a heterogeneous, multisystem disorder associated with a high risk of developing myelodysplastic syndrome (MDS) and the progression to acute myeloid leukemia. The mechanisms underlying malignant transformation in GATA2 deficiency remain poorly understood, necessitating predictive markers to assess an individual’s risk of progression and guide therapeutic decisions. In this study, we performed a systematic analysis of bone marrow biopsies from 57 pediatric MDS patients. Focusing on hematopoiesis and the hematopoietic niche, including its microenvironment, we used multiplex immunofluorescence combined with multispectral imaging, gene expression profiling, and multiplex RNA in situ hybridization. Patients with a GATA2 deficiency exhibited a dysregulated *GATA2* transcriptional network. Disease progression (GATA2-EB, *n* = 6) was associated with increased *GATA2* mRNA levels, restored expression of the GATA2 target *EZH2*, and increased H3K27me3. GATA2-EB was further characterized by the high expression of the anti-apoptotic protein BCL2, a feature absent in children with a GATA2 deficiency and refractory cytopenia of childhood (GATA2-RCC, *n* = 24) or other pediatric MDS subgroups (RCC, *n* = 17; MDS-EB, *n* = 10). The multispectral imaging analysis of additional BCL2 family members revealed significantly elevated Mediators of Apoptosis Combinatorial (MAC) scores in GATA2-EB patients. Taken together, our findings highlight the potential drivers of disease progression in GATA2 deficiency, particularly increased histone trimethylation and dysregulated apoptosis. Furthermore, upregulated BCL2 and *EZH2* and increased MAC scores provide a strong rationale for the use of venetoclax and azacitidine in therapeutic regimens for GATA2-EB.

## 1. Introduction

GATA2 deficiency is a complex and heterogeneous multisystem disorder with a broad spectrum of clinical and morphologic manifestations, encompassing conditions with overlapping characteristics such as Emberger syndrome, monocytopenia, and mycobacterial infections syndrome, or immune deficiency marked by monocytopenia, reduced dendritic cells, natural killer cells, and B lymphocytes [1,2,3,4]. Patients with a monoallelic germline mutation in *GATA2* present with hematologic complications early in life, accompanied by a significantly increased risk of developing myelodysplastic syndrome (MDS), which may progress to acute myeloid leukemia (AML) via a precarious malignant transformation [5,6,7]. In pediatric MDS, GATA2 deficiency is a prevalent disease entity, accounting for approximately 15% of advanced MDS cases [8,9,10]. Despite the identification of more than 300 variants in the *GATA2* gene [11], the association of specific mutations with the various clinical and histomorphologic phenotypes remains elusive.

In addition to the predisposition to myeloid neoplasms, GATA2 deficiency also leads to dysregulation of the hematopoietic niche and composition of the immune microenvironment [9,12]. Nevertheless, the extent of hematopoietic niche perturbation and its contribution to the development of MDS/AML remains largely unknown, making it difficult to draw reliable conclusions about disease progression. However, in order to facilitate the development of effective therapies, it is crucial to identify patients at risk of disease progression and malignant transformation at an early stage.

Therefore, the primary objective of this study was to elucidate the potential mechanisms or drivers underlying malignant transformation and to identify predictive markers that can help assess an individual’s risk of disease progression to guide therapeutic decisions and individualized patient management. To achieve this goal, we performed a comprehensive analysis of bone marrow biopsies from 57 children diagnosed with refractory cytopenia of childhood (RCC) or MDS with excess blasts (MDS-EB), with or without *GATA2* germline mutations, focusing on hematopoiesis and the hematopoietic niche, including the composition of its microenvironment.

## 2. Materials and Methods

### 2.1. Study Population

We studied 57 children with RCC and MDS-EB diagnosed according to the International Consensus Classification for pediatric MDS (Table 1) [13]. Analysis of the *GATA2* gene sequence, including intron 4, was conducted on bone marrow samples using targeted deep sequencing, with validation by Sanger sequencing. Subsequently, we confirmed the germline mutational status in non-myeloid tissues (fibroblasts). Patients were enrolled in the prospective studies of the European Working Group for MDS in Childhood (www.clinicaltrials.gov accessed on 25 October 2023; NCT00047268, NCT00662090) and two patients were referred to the Institute of Pathology, Lausanne University Hospital (CHUV), Switzerland. The study was approved by the Institutional Ethics Committee of the University of Freiburg (247/05). Informed consent was obtained from the patients and/or their legal guardians.

### 2.2. In Situ Hybridization

The RNAscope^®^ Multiplex Fluorescent Detection Kit v2 (ACD Bio-Techne, Minneapolis, MN, USA) was used to assess the mRNA expression of *EZH2*, *GATA2*, *IKZF1*, *LYL,1* and *RUNX1* and the co-localization of *CD34* and *EZH2* or *RUNX1*. In situ hybridization was performed using ready-to-use RNAscope^®^ probes (Hs-EZH2, Hs-GATA2, Hs-IKZF1, Hs-LYL1, and Hs-RUNX1, ACD Bio-Techne) or a combination of RNAscope^®^ probe Hs-CD34-C3 (ACD Bio-Techne) and Hs-EZH2 or Hs-RUNX1, following the manufacturer’s protocol. RNA probes were labeled with Opal™ fluorophores (Akoya Biosciences, Malborough, MA, USA).

### 2.3. Histological and Immunohistochemical Evaluation

Immunohistochemistry was performed to detect histone H3 trimethylation at lysine 27 (H3K27me3). After antigen retrieval, formalin-fixed paraffin-embedded (FFPE) bone marrow biopsies were incubated with antibodies against H3K27me3 (C36B11, 1:4000, Cell Signaling Technology, Danvers, MA, USA). The signal was visualized using the ImmPress anti-rabbit IgG polymer kit (Vector Laboratories, Burlingame, MA, USA) according to the manufacturer’s instructions. Semiquantitative scoring based on staining intensity and the percentage of positive cells was performed according to a standard immunoreactivity scoring system [14]. Immunoreactive scores were categorized as negative to weakly, moderately, and strongly positive.

### 2.4. Multiplex Immunofluorescence

Multiplex immunofluorescence assays were used for multispectral imaging of FFPE bone marrow specimen targeting H3K27me3 (C36B11, 1:100, Cell Signaling Technology, Danvers, MA, USA), CD34 (QEBnd-10, 1:100, Cell Marque, Darmstadt, Germany), BCL2 (124, 1:25, Agilent Dako, Santa Clara, CA, USA), MPO (polyclonal, 1:100, Agilent Dako, Santa Clara, CA, USA), BCL-XL (BX006+2H12, 1:75, Abcam, Cambridge, United Kingdom), and MCL1 (OTI10F6, 1:75, Thermo Fisher Scientific, Waltham, MA, USA). Antibodies were detected with Opal™ fluorophores (Akoya Biosciences, Malborough, MA, USA).

### 2.5. Gene Expression Profiling

RNA was purified from 10 µm thick FFPE bone marrow sections using the RNeasy^®^ DSP FFPE Kit (Qiagen, Hilden, Germany). RNA quality and quantity were assessed by spectrophotometry (Thermo Scientific™ NanoDrop™ One Spectrophotometer, Thermo Fisher Scientific, Waltham, MA, USA). Gene expression profiling was performed using the NanoString™ nCounter^®^ assay according to the manufacturer’s protocol (NanoString Technologies, Seattle, WA, USA). Briefly, 100 ng of purified RNA from seven representative patients was subjected to the profiling of 770 genes included in the nCounter^®^ Human PanCancer Immune Profiling Panel (NanoString Technologies, Seattle, WA, USA) (Table S1). Data were processed and analyzed using the nSolver™ 4.0 analysis software and the advanced analysis software plug-in version 2.0 (NanoString Technologies, Seattle, WA, USA).

### 2.6. Data Analysis and Visualization

Slides were scanned using the PhenoImager™ HT automated quantitative pathology imaging system (Akoya Biosciences, Malborough, MA, USA). Cells were quantified by the analysis of at least three regions of interest (ROIs) of 931 × 698 µm using the inForm^®^ automated image analysis software package (Akoya Biosciences, Malborough, MA, USA) and HALO^®^ 3.3, including HALO AI™ 3.3 (Indica Labs, Albuquerque, NM, USA).

### 2.7. Statistical Evaluation

GraphPad Prism, version 9.5.0 (GraphPad Software Inc., San Diego, CA, USA) was used for statistical analysis. The student’s *t*-test was used to evaluate continuous data. All *p*-values were two-tailed, with significance at *p* < 0.05.

## 3. Results

### 3.1. Patient Characteristics

We studied 57 children diagnosed with RCC or advanced MDS; 53% (30/57) of the pediatric patients in the cohort carried a germline *GATA2* mutation (GATA2^mut^), while the remaining 47% (27/57) were *GATA2* wild-type (GATA2^WT^). Among the GATA2^mut^ patients, 80% (24/30) presented with RCC and 20% (6/30) with MDS-EB. In the GATA2^WT^ group, MDS-EB accounted for 37% (10/27) of all cases. Their baseline characteristics are shown in Table 1.

Monosomy 7 was the predominant cytogenetic alteration in GATA2^mut^ patients (41%, 11/27; GATA2^mut^ RCC 5/21, and GATA2^mut^ MDS-EB 6/6) compared to the GATA2^WT^ cohort (30%, 8/27; GATA2^WT^ RCC 5/17, and GATA2^WT^ MDS-EB 3/10). A normal karyotype was more common in the GATA2^WT^ group than in the GATA2^mut^ group (56%, 15/27 in GATA2^WT^ versus 33%, 9/27 in GATA2^mut^, Table 1).

### 3.2. mRNA Expression of GATA2 and GATA2 Target Genes RUNX1, EZH2, IKZF1, and LYL1 Is Restored during Disease Progression in GATA2^mut^ Patients

**Figure 1:**
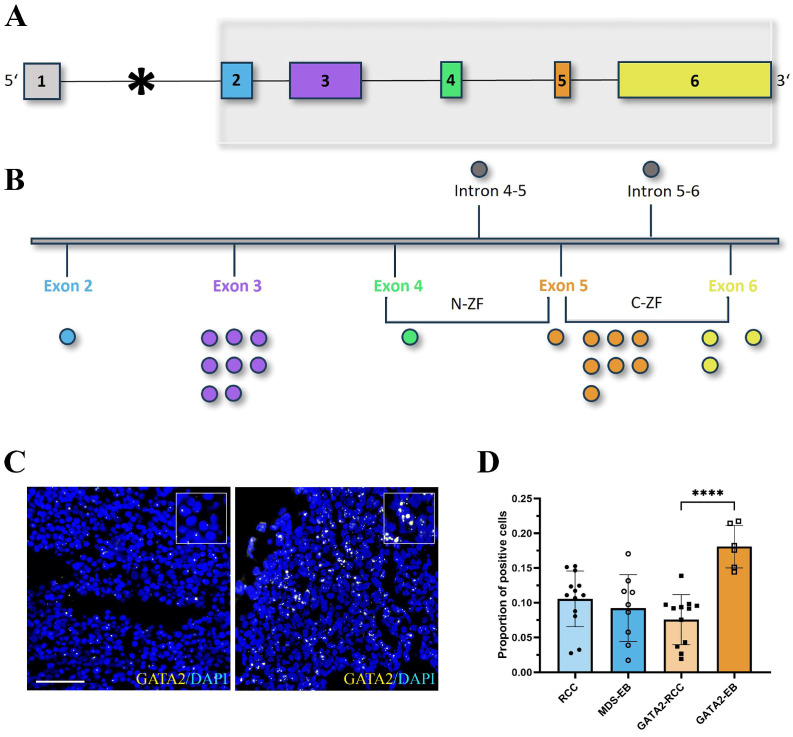
Localization of GATA2 mutations and GATA2 mRNA expression. Schematic overview of the genomic structure (**A**) of the *GATA2* gene, transcript variant 2 (NM_032638.5). Colored boxes represent exons 1-6. The asterisk indicates the binding site of the *GATA2* probe used for in situ hybridization in this study. Exons 2-6, highlighted in gray, are magnified (**B**) to illustrate the distribution of mutations identified in the cohort with respect to their localization within the gene. The binding site of the *GATA2* probe does not interfere with the localization of the different mutations on the gene. One mutation was identified in exon 2 (blue), eight mutations in exon 3 (purple), one mutation in exon 4 (green), within the N-zinc finger (N-ZF), one mutation in exon 5 (orange), between N-ZF and the C-zinc finger (C-ZF), seven mutations in exon 5, within C-ZF, two mutations in exon 6 (yellow), within C-ZF, and one mutation in exon 6, outside of C-ZF. One mutation was found in intron 4–5 and one in intron 5–6 (detailed mutation data Table S2). The in situ hybridization of *GATA2* (yellow) with DAPI counterstain (blue) is depicted in (**C**). A representative image of patients with a high mRNA signal is on the right and a low expression is on the left (scale bar = 25 µm). Quantification of *GATA2* mRNA expression (**D**) showed comparable proportions of GATA2-positive cells in patients with refractory cytopenia of childhood (RCC) and myelodysplastic syndrome with excess blasts (MDS-EB). In patients with a GATA2 deficiency, *GATA2* expression was significantly upregulated (**** *p* < 0.0001), with disease progression from RCC (GATA2-RCC) to MDS-EB (GATA2-EB).

To investigate the impact of GATA2 on the hematopoietic niche and its microenvironment, we performed in situ hybridization on GATA2^mut^ and GATA2^WT^ patients. First, the mRNA expression of *GATA2* itself was determined. Importantly, the binding site of the *GATA2* probe used in this study was designed to avoid interference with previously reported mutations in GATA2 deficiency [8,10], as well as with distinct mutations found in our cohort of GATA2^mut^ patients (Figure 1A,B; detailed mutational data Table S2). Thus, the detected expression reflects the amount of *GATA2* mRNA derived from both alleles.

Consistent with previous studies documenting haploinsufficiency and dysregulation of gene expression following *GATA2* germline mutations [1,5,8], a marked decrease in *GATA2* mRNA expression was detected in GATA2^mut^ patients with RCC (GATA2-RCC) (Figure 1C,D). Notably, within our cohort, we observed a significant increase in *GATA2* expression during disease progression in GATA2^mut^ patients with excess blasts (GATA2-EB) (mean ± standard deviation (SD) in GATA2-EB 0.1808 ± 0.03071 versus 0.07559 ± 0.03603 in GATA2-RCC, *p* < 0.0001). This effect was not present among patients without a GATA2 deficiency and *GATA2* mRNA expression remained stable during disease progression (mean ± SD 0.09223 ± 0.04805 in MDS-EB and 0.1055 ± 0.04004 in RCC, Figure 1D).

**Figure 2:**
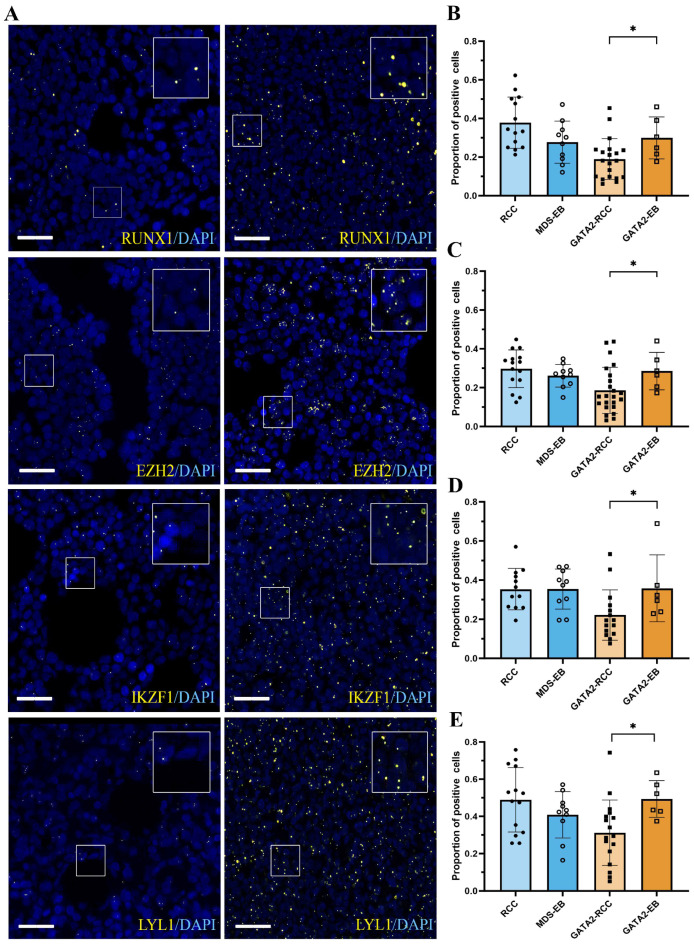
Increase in mRNA expression of *RUNX1*, *EZH2*, *IKZF1*, and *LYL1* with disease progression and a GATA2 deficiency. Overview of in situ hybridization (**A**) for the GATA2 target genes *RUNX1*, *EZH2*, *IKZF1*, and *LYL1* (yellow). Representative images for patients with a low mRNA signal on the left and a strong expression on the right with DAPI counterstain (blue). The scale bar corresponds to 25 µm. Quantification of mRNA expression for *RUNX1* (**B**), *EZH2* (**C**), *IKZF1* (**D**), and *LYL1* (**E**) showing comparable expression levels of positive cells in RCC and MDS-EB patients without a GATA2 deficiency. In patients with a GATA2 deficiency, the expression of all four GATA2 targets increased significantly (* *p* < 0.05) with disease progression.

To further characterize the downstream effects of *GATA2* mRNA upregulation, in situ hybridization was performed on the four genes regulated by GATA2 (*RUNX1*, *EZH2*, *IKZF1*, and *LYL1*) (Figure 2A). Interestingly, GATA2-EB patients displayed a significantly increased expression of all four GATA2 targets compared to the GATA2-RCC group (*p* < 0.05, Figure 2B–E). In contrast, the expression levels of *RUNX1*, *EZH2*, *IKZF1*, and *LYL1* did not differ significantly between RCC and MDS-EB patients in the GATA2^WT^ group (Figure 2B–E).

Since monosomy 7 is the predominant cytogenetic abnormality observed in our cohort of GATA2^mut^ patients, the effect of this karyotype on the expression of GATA2 target genes was evaluated separately. The results for patients with monosomy 7 were not significantly different from those with other cytogenetic lesions (Figure S1A–D). While monosomy 7 has been associated with different hematologic phenotypes in peripheral blood samples of GATA2^mut^ and GATA2^WT^ patients [8], our findings in the bone marrow specimens did not support this association at the mRNA level.

### 3.3. In GATA2 Haploinsufficient Patients, EZH2 Expression Is Increased in Hematopoietic Progenitors at Advanced Disease Stage

**Figure 3:**
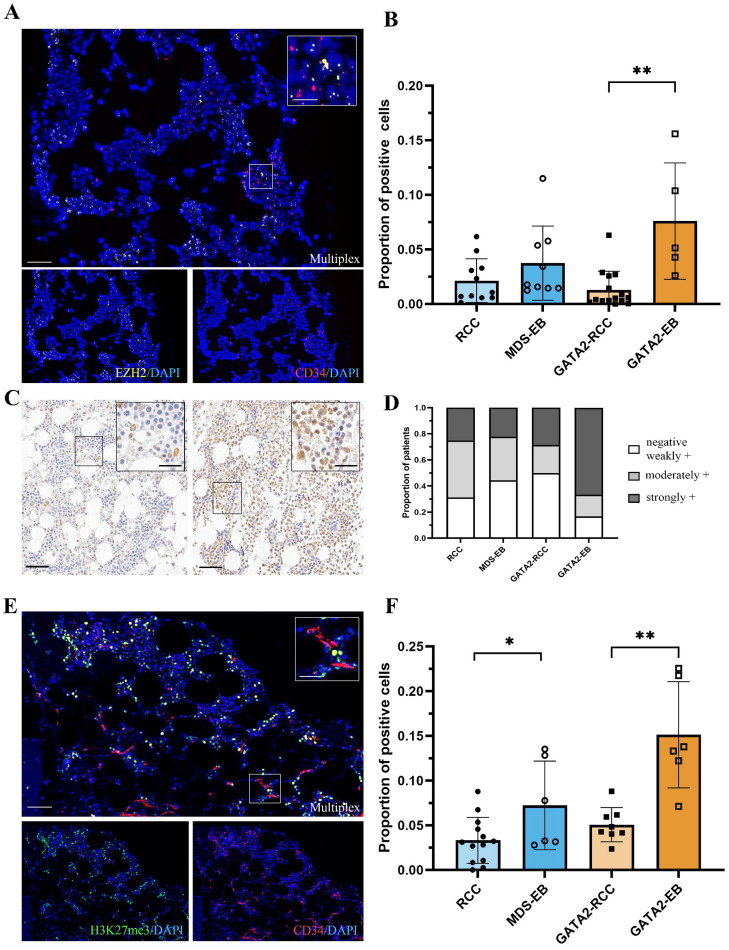
Increased *EZH2* expression of hematopoietic progenitors associated with upregulated histone H3 trimethylation at lysine 27 (H3K27me3) during advanced disease in patients with a GATA2 deficiency. Multiplex in situ hybridization (**A**) of *EZH2* (yellow) and *CD34* (red) with DAPI counterstain (blue). An overview (scale bar = 100 µm) and magnified inlet (scale bar = 50 µm) are shown. Quantification of *EZH2*-positive hematopoietic progenitors (**B**) with an increased number in GATA2-EB patients (** *p* < 0.01). Overview of immunohistochemistry (**C**) for H3K27me3 representing strong (right) and weak (left) histone trimethylation (brown), and hematoxylin counterstain (blue). Overview images (scale bar = 100 µm) and magnified inlets (scale bar = 50 µm) are shown. Immunoreactive score (**D**): Most GATA2-EB patients were strongly positive for H3K27me3, whereas MDS-EB and GATA2-RCC were predominantly negative/weakly positive and RCC moderately positive. Multiplex immunofluorescence (**E**) for CD34 (red) and H3K27me3 (green) with DAPI counterstaining (blue). The scale bar is 100 µm for the overview image and 50 µm for the magnified inlet. Quantification of the H3K27me3-positive hematopoietic progenitors (**F**) showed a strong upregulation in hematopoietic progenitors expressing H3K27me3 (** *p* < 0.01) in patients with a GATA2 deficiency and disease progression compared to patients without the *GATA2* mutation (* *p* < 0.05).

To explore whether the changes in the *GATA2* transcriptional network extend to the hematopoietic stem cell pool, we performed combinatorial in situ hybridization of *CD34* and *RUNX1* (Figure S2A) or *EZH2* (Figure 3A). Remarkably, upregulated levels of *EZH2*-positive hematopoietic progenitors were observed in GATA2-EB compared to GATA2-RCC patients (the proportion of *EZH2*-positive progenitors mean ± SD in GATA2-EB was 0.07596 ± 0.05328 versus 0.01293 ± 0.01708 in GATA2-RCC, *p* < 0.01). In contrast, patients without a GATA2 deficiency did not show this effect (Figure 3B). Notably, the expression of *RUNX1*-positive hematopoietic progenitors did not vary significantly across all four patient groups, regardless of the presence of excess blasts in the advanced disease stages MDS-EB and GATA2-EB (mean ± SD in GATA2-RCC was 0.01459 ± 0.02384, GATA2-EB was 0.01924 ± 0.01474, RCC was 0.01806 ± 0.02900, and MDS-EB was 0.02243 ± 0.03122, Figure S2B). Thus, the increased *EZH2* expression in the hematopoietic progenitors of GATA2-EB patients cannot be attributed solely to an increased blast count.

### 3.4. Robust Expression of the EZH2-Dependent H3K27me3 Gene Silencing Mark in GATA2-EB Patients

Since *EZH2* plays a critical role in the epigenetic silencing of gene transcription and catalyzes the trimethylation of histone H3 at lysine 27 (H3K27me3) [15,16], we next sought to determine the presence of this gene silencing mark in our study cohort (Figure 3C). Semiquantitative scoring of immunohistochemical staining for H3K27me3 revealed that most GATA2-EB patients were strongly positive for H3K27me3 (66.66% of GATA2-EB, Figure 3D). In addition, the immunoreactivity scores for GATA2-RCC and MDS-EB patients were predominantly negative to weakly positive (50% of GATA2-RCC and 44.45% of MDS-EB), with RCC patients showing moderately positive scores (43.75% of RCC, Figure 3D).

To further validate the increased levels of *EZH2*-positive hematopoietic progenitors observed in GATA2-EB patients, we performed multiplex immunofluorescence for CD34 and H3K27me3 (Figure 3E). Consistent with these findings, we observed an increase in hematopoietic progenitors expressing H3K27me3 in GATA2-EB patients compared to GATA2-RCC patients (the proportion of H3K27me3-positive progenitors mean ± SD in GATA2-EB was 0.1512 ± 0.05944 versus 0.05063 ± 0.01921 in GATA2-RCC, *p* < 0.01, Figure 3F). A higher number of H3K27me3-positive hematopoietic progenitor cells was also found in the MDS-EB group compared to RCC, although the effect was less pronounced when compared to GATA2^mut^ patients (MDS-EB 0.07221 ± 0.04964 versus RCC 0.03315 ± 0.02572, *p* < 0.05).

### 3.5. GATA2^mut^ Patients Show Increased BCL2 Expression with Disease Progression

**Figure 4:**
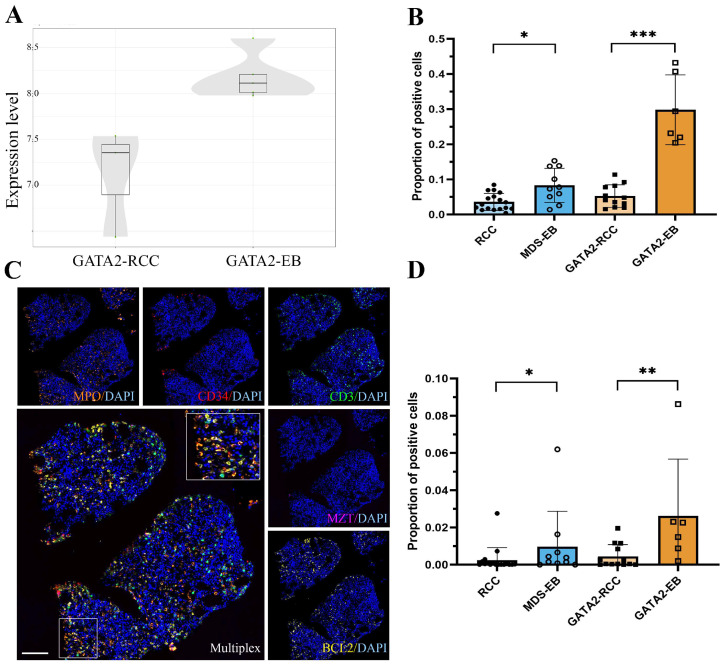
Elevated anti-apoptotic signaling with disease progression in patients with a GATA2 deficiency. Box and whisker plot (**A**) for the differential gene expression of *BCL2* in GATA2-EB versus GATA2-RCC patients. The plot shows the median expression along with the lower and upper quartiles at the box minima and maxima, respectively. The normalized *BCL2* expression level is upregulated in the GATA2-EB group. Quantification of BCL2-positive cells from multiplex immunofluorescence (**B**) corroborated the gene expression profiling data at the protein level, with strongly increasing BCL2 expression during disease progression in patients with a GATA2 deficiency (*** *p* < 0.001) compared to patients without the *GATA2* mutation (* *p* < 0.05). Multiplex immunofluorescence images (**C**) for profiling the BCL2 expression in key bone marrow cell populations consisting of myeloid cells (MPO, orange), hematopoietic progenitor cells (CD34, red), T lymphocytes (CD3, green), and mast cells (MZT, pink). The scale bar corresponds to 100 µm. The quantification of MPO-positive cells co-expressing BCL2 (**D**) showed increased levels of BCL2-expressing myeloid cells in GATA2-EB patients (** *p* < 0.01), with lower levels during disease progression in patients without a GATA2 deficiency (* *p* < 0.05).

To further characterize the hematopoietic niche in GATA2 deficiency, we performed transcriptome analysis on the representative samples from GATA2-RCC and GATA2-EB patients (data on the 15 most differentially expressed genes in Table S3). Strikingly, a strong differential expression of the anti-apoptotic *BCL2* was identified in the GATA2^mut^ patient samples, with higher expression levels observed in the GATA2-EB group (Figure 4A). In support of this observation, multiplex immunofluorescence revealed a marked increase in BCL2 protein expression, specifically in GATA2-EB patients, compared to all other patient groups (the proportion of BCL2-positive cells mean ± SD in GATA2-EB was 0. 2980 ± 0.09923 versus 0.05252 ± 0.03191 in GATA2-RCC, *p* < 0.001; MDS-EB was 0.08308 ± 0.04836 versus RCC 0.03574 ± 0.02431, *p* < 0.05, Figure 4B).

To further elucidate the cell types exhibiting upregulated BCL2 expression, we used multiplex immunofluorescence to profile key cell populations within the bone marrow. Our analysis revealed an increased number of BCL2-expressing myeloid cells in GATA2-EB patients (Figure 4C,D). In patients without a GATA2 deficiency, the myeloid cell population showed a comparatively lower level of BCL2 during disease progression.

### 3.6. Profiling of BCL2 Family Members Reveals an Increased MAC Score in GATA2-EB Patients

**Figure 5:**
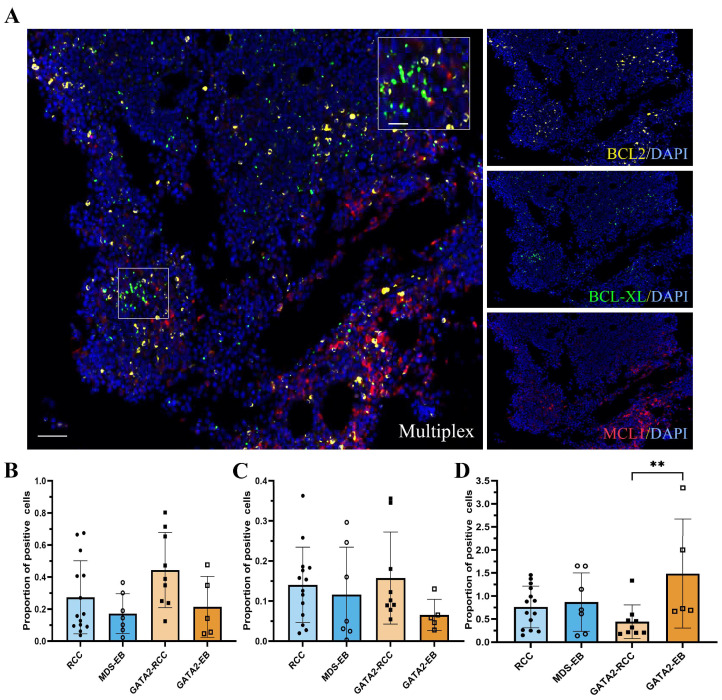
Increased Mediators of Apoptosis Combinatorial (MAC) score in GATA2-EB patients. Multiplex immunofluorescence (**A**) for BCL-XL (green), BCL2 (yellow), and MCL1 (red); DAPI counterstain (blue) (scale bar = 100 µm, magnified inlet 50 µm). The quantification of MCL1 (**B**) and BCL-XL (**C**) exhibited a trend with reduced levels of both BCL2 family proteins in patients with a GATA2 deficiency and advanced MDS. Multiplex immunofluorescence showed an upregulated MAC score (** *p* < 0.01) (**D**) in GATA2-EB patients compared to GATA2-RCC and patients without a GATA2 deficiency.

In addition to BCL2, its homologs MCL1 and BCL-XL orchestrate cellular survival by binding and inhibiting pro-survival BCL-2 proteins [17,18,19]. Consistent with the literature [17,18,19], our analysis revealed an opposite trend in the expression levels of MCL1 and BCL-XL compared to BCL2, with a reduction in both BCL2 family members in GATA2-EB patients, although not reaching statistical significance (Figure 5A–C). In the RCC and MDS-EB groups, MCL1 and BCL-XL showed relatively comparable expression levels.

To integrate the protein expression of BCL2, MCL1, and BCL-XL into a single score, we adopted the previously reported flow cytometry-based MAC score methodology [20]. We calculated the MAC score by multiplex immunofluorescence using the following formula:

$$\left[MAC\;score=\frac{BCL2^{+}cells}{MCL1^{+}cells+BCL-XL^{+}cells}\right].$$

Patients with a GATA2 deficiency showed significantly elevated MAC scores in advanced disease (MAC score mean ± SD in GATA2-EB was 1.487 ± 1.184 versus 0.4425 ± 0.3659 in GATA2-RCC, *p* < 0.01, Figure 5D). In contrast, the MAC score of patients without a GATA2 deficiency did not show any notable change with disease progression (MAC score mean ± SD in MDS-EB of 0.8686 ± 0.6336 versus 0.7621 ± 0.4522 in RCC).

In addition, we quantified the number of single positive cells (BCL2^+^, MCL1^−^, and BCL-XL^−^) for all bone marrow samples. Patients with RCC and MDS-EB showed a comparable expression of BCL2 single positive cells (mean ± SD in RCC of 0.08822 ± 0.05149 and in MDS-EB, 0.1195 ± 0.1021). In contrast, patients with a GATA2 deficiency exhibited a significant upregulation in (BCL2^+^, MCL1^−^, and BCL-XL^−^) cells with disease progression (mean ± SD in GATA2-EB of 0.1878 ± 0.1565 versus 0.06026 ± 0.08405 in GATA2-RCC, *p* < 0.05).

## 4. Discussion

The complex mechanisms and underlying drivers leading to malignant transformation in pediatric GATA2 deficiency are currently poorly understood, highlighting the urgent need for predictive markers to help assess the individual risk of disease progression and guide therapeutic decisions.

In this study, we demonstrate a dysregulation of the *GATA2* transcriptional network in GATA2^mut^ patients. Among individuals with GATA2-RCC, a marked reduction in *GATA2* mRNA expression was evident. This observation resonates with previous studies that highlighted haploinsufficiency and disruption in gene expression resulting from germline mutations within *GATA2* [1,5,8]. As expected, the four investigated genes *RUNX1*, *EZH2*, *IKZF1*, and *LYL1*, all under the regulatory control of GATA2 [21], were also downregulated in GATA2^mut^ patients with RCC. Interestingly, during disease progression, patients exhibited restored expression associated with upregulation in *GATA2*, an observation previously not reported in the scientific literature. This resurgence of *GATA2*-associated expression dynamics during disease progression underscores a novel facet of the disease trajectory.

Taking into account the well-established role of the histone methyltransferase *EZH2* in promoting the transformation of adult MDS [22], we focused our attention on an in-depth analysis of *EZH2* within our patient cohort. Double in situ hybridization revealed an upregulation in histone methyltransferase *EZH2* in hematopoietic (CD34-positive) progenitor cells in GATA2-EB. Given the pivotal role of *EZH2* in orchestrating epigenetic silencing by catalyzing the trimethylation of histone H3 at lysine 27 [15,16], we then analyzed the expression pattern of H3K27me3 in our patient cohort. Indeed, the GATA2-EB bone marrow biopsies were strongly positive for this gene silencing mark, whereas GATA2-RCC or non-GATA2 biopsies were negative or only weakly positive. Combining in situ hybridization with multiplex immunofluorescence revealed that CD34-positive progenitors were, in fact, positive for H3K27me3. In conclusion, we observed increased levels of *EZH2* associated with increased histone trimethylation H3K27me3 in the disease progression of patients with a GATA2 deficiency.

These results underscore the pivotal role that *EZH2* plays in orchestrating histone trimethylation, mirroring observations previously documented in the context of adult AML/MDS patients [23,24,25] and highlighting its potential as a prognostically relevant marker in myeloid neoplasms. Furthermore, while the influence of *EZH2* and epigenetics in MDS has been extensively studied in adults [23,26,27,28,29], our findings of robust *EZH2* expression associated with increased histone trimethylation in pediatric GATA2-EB patients contribute to the growing understanding of the influence of epigenetic factors on disease progression in children [30].

In addition, through our comprehensive analysis, we identified a significant increase in the expression of the anti-apoptotic protein BCL2 within the myeloid cell population of patients with a GATA2 deficiency during disease progression. This compelling finding underscores the emergence of acquired resistance to apoptotic cell death, a central mechanism in cellular homeostasis. While the dysregulation of apoptosis signaling and the subsequent development of increased apoptotic resistance in high-risk MDS is well-established in adults [31,32,33,34], evidence is lacking for pediatric MDS patients. The present study, however, serves to fill this knowledge gap by postulating that the orchestrated upregulation in anti-apoptotic pathways may potentially play a pivotal role in disease progression in pediatric patients with a GATA2 deficiency. This finding not only advances our understanding of the underlying pathogenic processes but also provides a critical link between adult and pediatric cases of the disorder, thus contributing to a more holistic understanding of its complex etiology.

Alongside BCL2, the BCL2 family members MCL1 and BCL-XL are known to promote cell survival [17,18,19]. The MAC score, proposed by Waclawiczek et al. [20], uses the combined expression of BCL2 family members BCL2, MCL1, and BCL-XL to predict the response to BCL2-inhibitor venetoclax (VEN) and DNA hypomethylating agent azacitidine (AZA) combination therapy in AML patients. In our patient cohort, the MAC scores of GATA2-EB patients were significantly increased compared to the GATA2-RCC group. Conversely, patients without a GATA2 deficiency did not show a significant change in their MAC score with disease progression. The elevated MAC scores observed in GATA2-EB patients suggest a potential positive response to VEN/AZA combination therapy. In addition, our study revealed robust expression levels of BCL2 and *EZH2* in GATA2-EB patients. BCL2 is directly targeted by VEN and the increased *EZH2* expression, accompanied by enhanced histone trimethylation, highlights the importance of epigenetic regulation in GATA2-EB. Thus, these results support the potential use of epigenetic agents such as AZA to effectively target epigenetic alterations in MDS. Taken together, these findings strongly suggest that GATA2-EB patients may benefit from treatment regimens that include VEN and AZA, either alone or in combination.

While this study provides valuable insights into the dynamics of malignant transformation in GATA2 deficiency, a rare disease in children, and its potential therapeutic implications, it is important to consider the small sample size of six patients in the GATA2-EB group, which may have some impact on statistical power. We addressed this by including a comparison group of 10 MDS-EB patients without a GATA2 deficiency to serve as a valuable reference for the analysis. The fact that the effects observed in the GATA2-EB group were not replicated in MDS-EB patients strengthens the clear distinction between patients with and without a GATA2 deficiency. However, it is important to acknowledge that the small group sizes may slightly affect the estimation of the true effect size, suggesting the need for further investigation in larger cohorts.

Our study used a multimodal approach, integrating a comprehensive examination of the hematopoietic niche and its microenvironment with transcriptome analysis. By integrating multiple data sources, this approach allows us to provide a foundation for understanding the potential therapeutic benefits of VEN and AZA individually, as well as their combined efficacy as predicted by the MAC score. Building on the extensively studied efficacy of VEN and AZA in adult AML/MDS patients [35,36,37,38], our research addresses the limited evidence regarding their use in the pediatric population. Recent retrospective studies have provided initial indications of the safety and potential benefits of VEN-based regimens, including the combination of VEN/AZA, in pediatric patients with advanced MDS [39,40]. In this context, the present study contributes significantly to these findings by providing additional evidence supporting the use of VEN and AZA in childhood MDS, particularly highlighting their potential benefits in patients with a GATA2 deficiency and an advanced disease stage.

## 5. Conclusions

In conclusion, we identified increased histone trimethylation and deregulated apoptosis as the potential drivers of malignant transformation in patients with a GATA2 deficiency. These findings provide valuable insights into the potential underlying mechanisms of disease progression in GATA2^mut^ patients and have implications for tailoring personalized therapeutic strategies. In particular, our results suggest that treatment regimens incorporating VEN and AZA, either individually or in combination, hold promise for GATA2-EB patients.

## Figures and Tables

**Table 1:** Patient characteristics.

Parameter		GATA2^mut^ *n* = 30	GATA2^WT^ *n* = 27
Age at onset	Years, median (range)	13 (4–20)	9 (2–19)
Sex	Males, *n* (%)	16 (53)	16 (59)
	Females, *n* (%)	14 (47)	11 (41)
Diagnosis	RCC, *n* (%)	24 (80)	17 (63)
	MDS-EB, *n* (%)	6 (20)	10 (37)
	Normal, *n* (%)	9 (33)	15 (56)
Karyotype ^1^	Monosomy 7, *n* (%) ^2^	11 (41)	8 (30)
	Trisomy 8, *n* (%)	7 (26)	3 (11)
	Other ^3^, *n* (%)	0 (0)	1 (3)

^1^ no information available for *n* = 3 children in the GATA2mut group; ^2^ includes monosomy 7 with additional mutations in *ASXL1* and *NF1*; ^3^ random aberration; GATA2^mut^: *GATA2* mutation; GATA2^WT^: *GATA2* wild-type; MDS-EB: myelodysplastic syndrome with excess blasts; and RCC: refractory cytopenia of childhood.

## Data Availability

All data supporting the findings of this study are included within this article or its supplementary material.

## Supplementary Materials

The following supporting information can be downloaded at: https://www.mdpi.com/article/10.3390/cancers15235594/s1, Table S1: NanoString nCounter^®^ Human PanCancer Immune Profiling Panel; Table S2: Distinct mutations found in pediatric patients with a GATA2 deficiency and their localization on the *GATA2* gene; Table S3: Gene expression data for the 15 most differentially expressed genes in patients with a GATA2 deficiency in early and advanced disease stages in alphabetical order; Figure S1: Impact of the karyotype of patients on the expression of GATA2 target genes; Figure S2: Comparable expression of *RUNX1*-positive hematopoietic progenitors in all patient groups, regardless of their *GATA2* mutational status and disease stage.
