# Supplementary material for: Development of MDS in Pediatric Patients with GATA2 Deficiency: Increased Histone Trimethylation and Deregulated Apoptosis as Potential Drivers of Transformation
Source: Cancers (Basel). 2023 Nov 26;15(23):5594. doi: 10.3390/cancers15235594 (PMC10705137; doi:10.3390/cancers15235594)
Supplement: Supplementary file 1 [file cancers-15-05594-s001.zip › cancers-2713053-supplementary.pdf]

Gene name	Type	ProbeID
A2M	Probe	NM_000014.4
ABCB1	Probe	NM_000927.3
ABL1	Probe	NM_005157.3
ADA	Probe	NM_000022.2
ADORA2A	Probe	NM_000675.3
AICDA	Probe	NM_020661.1
AIRE	Probe	NM_000383.2
AKT3	Probe	NM_181690.1
ALCAM	Probe	NM_001627.3
AMBP	Probe	NM_001633.3
AMICA1	Probe	NM_153206.2
ANP32B	Probe	NM_006401.2
ANXA1	Probe	NM_000700.1
APOE	Probe	NM_000041.2
APP	Probe	NM_000484.3
ARG1	Probe	NM_000045.2
ARG2	Probe	NM_001172.3
ATF1	Probe	NM_005171.2
ATF2	Probe	NM_001256090.1
ATG10	Probe	NM_001131028.1
ATG12	Probe	NM_004707.2
ATG16L1	Probe	NM_198890.2
ATG5	Probe	NM_004849.2
ATG7	Probe	NM_001136031.2
ATM	Probe	NM_000051.3
AXL	Probe	NM_021913.2
BAGE	Probe	NM_001187.1
BATF	Probe	NM_006399.3
BAX	Probe	NM_138761.3
BCL10	Probe	NM_003921.2
BCL2	Probe	NM_000657.2
BCL2L1	Probe	NM_001191.2
BCL6	Probe	NM_001706.2
BID	Probe	NM_001196.2
BIRC5	Probe	NM_001168.2
BLK	Probe	NM_001715.2
BLNK	Probe	NM_013314.2
BMI1	Probe	NM_005180.5
BST1	Probe	NM_004334.2
BST2	Probe	NM_004335.2
BTK	Probe	NM_000061.1
BTLA	Probe	NM_181780.2
C1QA	Probe	NM_015991.2
C1QB	Probe	NM_000491.3
C1QBP	Probe	NM_001212.3

C1R	Probe	NM_001733.4
C1S	Probe	NM_001734.2
C2	Probe	NM_000063.3
C3	Probe	NM_000064.2
C3AR1	Probe	NM_004054.2
C4B	Probe	NM_001002029.3
C4BPA	Probe	NM_000715.3
C5	Probe	NM_001735.2
C6	Probe	NM_000065.2
C7	Probe	NM_000587.2
C8A	Probe	NM_000562.2
C8B	Probe	NM_000066.2
C8G	Probe	NM_000606.2
C9	Probe	NM_001737.3
CAMP	Probe	NM_004345.3
CARD11	Probe	NM_032415.2
CARD9	Probe	NM_052813.4
CASP1	Probe	NM_001223.3
CASP10	Probe	NM_032977.3
CASP3	Probe	NM_032991.2
CASP8	Probe	NM_001228.4
CCL1	Probe	NM_002981.1
CCL11	Probe	NM_002986.2
CCL13	Probe	NM_005408.2
CCL14	Probe	NM_032963.3
CCL15	Probe	NM_032965.3
CCL16	Probe	NM_004590.2
CCL17	Probe	NM_002987.2
CCL18	Probe	NM_002988.2
CCL19	Probe	NM_006274.2
CCL2	Probe	NM_002982.3
CCL20	Probe	NM_004591.1
CCL21	Probe	NM_002989.2
CCL22	Probe	NM_002990.3
CCL23	Probe	NM_145898.1
CCL24	Probe	NM_002991.2
CCL25	Probe	NM_005624.2
CCL26	Probe	NM_006072.4
CCL27	Probe	NM_006664.2
CCL28	Probe	NM_148672.2
CCL3	Probe	NM_002983.2
CCL3L1	Probe	NM_021006.4
CCL4	Probe	NM_002984.2
CCL5	Probe	NM_002985.2
CCL7	Probe	NM_006273.2
CCL8	Probe	NM_005623.2

CCND3	Probe	NM_001760.2
CCR1	Probe	NM_001295.2
CCR2	Probe	NM_001123041.2
CCR3	Probe	NM_001837.2
CCR4	Probe	NM_005508.4
CCR5	Probe	NM_000579.1
CCR6	Probe	NM_031409.2
CCR7	Probe	NM_001838.2
CCR9	Probe	NM_031200.1
CCRL2	Probe	NM_003965.4
CD14	Probe	NM_000591.2
CD160	Probe	NM_007053.2
CD163	Probe	NM_004244.4
CD164	Probe	NM_006016.4
CD180	Probe	NM_005582.2
CD19	Probe	NM_001770.4
CD1A	Probe	NM_001763.2
CD1B	Probe	NM_001764.2
CD1C	Probe	NM_001765.2
CD1D	Probe	NM_001766.3
CD1E	Probe	NM_001042583.1
CD2	Probe	NM_001767.3
CD200	Probe	NM_005944.5
CD207	Probe	NM_015717.2
CD209	Probe	NM_021155.2
CD22	Probe	NM_001771.2
CD24	Probe	NM_013230.2
CD244	Probe	NM_016382.2
CD247	Probe	NM_198053.1
CD27	Probe	NM_001242.4
CD274	Probe	NM_014143.3
CD276	Probe	NM_001024736.1
CD28	Probe	NM_001243078.1
CD33	Probe	NM_001177608.1
CD34	Probe	NM_001025109.1
CD36	Probe	NM_001001548.2
CD37	Probe	NM_001774.2
CD38	Probe	NM_001775.2
CD3D	Probe	NM_000732.4
CD3E	Probe	NM_000733.2
CD3EAP	Probe	NM_012099.1
CD3G	Probe	NM_000073.2
CD4	Probe	NM_000616.4
CD40	Probe	NM_001250.4
CD40LG	Probe	NM_000074.2
CD44	Probe	NM_001001392.1

CD46	Probe	NM_172350.1
CD47	Probe	NM_001777.3
CD48	Probe	NM_001778.2
CD5	Probe	NM_014207.2
CD53	Probe	NM_001040033.1
CD55	Probe	NM_000574.3
CD58	Probe	NM_001779.2
CD59	Probe	NM_000611.4
CD6	Probe	NM_006725.3
CD63	Probe	NM_001780.4
CD68	Probe	NM_001251.2
CD7	Probe	NM_006137.6
CD70	Probe	NM_001252.2
CD74	Probe	NM_001025159.1
CD79A	Probe	NM_001783.3
CD79B	Probe	NM_021602.2
CD80	Probe	NM_005191.3
CD81	Probe	NM_004356.3
CD83	Probe	NM_004233.3
CD84	Probe	NM_001184879.1
CD86	Probe	NM_175862.3
CD8A	Probe	NM_001768.5
CD8B	Probe	NM_004931.3
CD9	Probe	NM_001769.2
CD96	Probe	NM_005816.4
CD97	Probe	NM_078481.2
CD99	Probe	NM_002414.3
CDH1	Probe	NM_004360.2
CDH5	Probe	NM_001795.3
CDK1	Probe	NM_001786.4
CDKN1A	Probe	NM_000389.2
CEACAM1	Probe	NM_001712.3
CEACAM6	Probe	NM_002483.4
CEACAM8	Probe	NM_001816.3
CEBPB	Probe	NM_005194.2
CFB	Probe	NM_001710.5
CFD	Probe	NM_001928.2
CFI	Probe	NM_000204.3
CFP	Probe	NM_002621.2
CHIT1	Probe	NM_003465.2
CHUK	Probe	NM_001278.3
CKLF	Probe	NM_181640.2
CLEC4A	Probe	NM_194448.2
CLEC4C	Probe	NM_203503.1
CLEC5A	Probe	NM_013252.2
CLEC6A	Probe	NM_001007033.1

CLEC7A	Probe	NM_197954.2
CLU	Probe	NM_001831.2
CMA1	Probe	NM_001836.2
CMKLR1	Probe	NM_004072.1
COL3A1	Probe	NM_000090.3
COLEC12	Probe	NM_130386.2
CR1	Probe	NM_000651.4
CR2	Probe	NM_001006658.1
CREB1	Probe	NM_004379.3
CREB5	Probe	NM_182898.2
CREBBP	Probe	NM_004380.2
CRP	Probe	NM_000567.2
CSF1	Probe	NM_000757.4
CSF1R	Probe	NM_005211.2
CSF2	Probe	NM_000758.2
CSF2RB	Probe	NM_000395.2
CSF3	Probe	NM_000759.3
CSF3R	Probe	NM_156038.2
CT45A1	Probe	NM_001017417.1
CTAG1B	Probe	NM_001327.2
CTAGE1	Probe	NM_172241.2
CTCFL	Probe	NM_001269042.1
CTLA4	Probe	NM_005214.3
CTSG	Probe	NM_001911.2
CTSH	Probe	NM_004390.3
CTSL	Probe	NM_001912.4
CTSS	Probe	NM_004079.3
CTSW	Probe	NM_001335.3
CX3CL1	Probe	NM_002996.3
CX3CR1	Probe	NM_001337.3
CXCL1	Probe	NM_001511.1
CXCL10	Probe	NM_001565.1
CXCL11	Probe	NM_005409.4
CXCL12	Probe	NM_000609.5
CXCL13	Probe	NM_006419.2
CXCL14	Probe	NM_004887.4
CXCL16	Probe	NM_001100812.1
CXCL2	Probe	NM_002089.3
CXCL3	Probe	NM_002090.2
CXCL5	Probe	NM_002994.3
CXCL6	Probe	NM_002993.3
CXCL9	Probe	NM_002416.1
CXCR1	Probe	NM_000634.2
CXCR2	Probe	NM_001557.2
CXCR3	Probe	NM_001504.1
CXCR4	Probe	NM_003467.2

CXCR5	Probe	NM_001716.3
CXCR6	Probe	NM_006564.1
CYBB	Probe	NM_000397.3
CYFIP2	Probe	NM_001037332.2
CYLD	Probe	NM_015247.1
DDX43	Probe	NM_018665.2
DDX58	Probe	NM_014314.3
DEFB1	Probe	NM_005218.3
DMBT1	Probe	NM_007329.2
DOCK9	Probe	NM_001130048.1
DPP4	Probe	NM_001935.3
DUSP4	Probe	NM_057158.2
DUSP6	Probe	NM_001946.2
EBI3	Probe	NM_005755.2
ECSIT	Probe	NM_001142464.2
EGR1	Probe	NM_001964.2
EGR2	Probe	NM_000399.3
ELANE	Probe	NM_001972.2
ELK1	Probe	NM_005229.3
ENG	Probe	NM_001114753.1
ENTPD1	Probe	NM_001098175.1
EOMES	Probe	NM_005442.2
EP300	Probe	NM_001429.2
EPCAM	Probe	NM_002354.1
ETS1	Probe	NM_005238.3
EWSR1	Probe	NM_013986.3
F12	Probe	NM_000505.3
F13A1	Probe	NM_000129.3
F2RL1	Probe	NM_005242.3
FADD	Probe	NM_003824.2
FAS	Probe	NM_000043.3
FCER1A	Probe	NM_002001.2
FCER1G	Probe	NM_004106.1
FCER2	Probe	NM_002002.4
FCGR1A	Probe	NM_000566.3
FCGR2A	Probe	NM_021642.3
FCGR2B	Probe	NM_001002273.1
FCGR3A	Probe	NM_000569.6
FEZ1	Probe	NM_005103.4
FLT3	Probe	NM_004119.1
FLT3LG	Probe	NM_001459.3
FN1	Probe	NM_212482.1
FOS	Probe	NM_005252.2
FOXJ1	Probe	NM_001454.3
FOXP3	Probe	NM_014009.3
FPR2	Probe	NM_001462.3

FUT5	Probe	NM_002034.2
FUT7	Probe	NM_004479.3
FYN	Probe	NM_002037.3
GAGE1	Probe	NM_001040663.2
GATA3	Probe	NM_001002295.1
GNLY	Probe	NM_006433.2
GPI	Probe	NM_000175.2
GTF3C1	Probe	NM_001520.3
GZMA	Probe	NM_006144.2
GZMB	Probe	NM_004131.3
GZMH	Probe	NM_033423.3
GZMK	Probe	NM_002104.2
GZMM	Probe	NM_005317.2
HAMP	Probe	NM_021175.2
HAVCR2	Probe	NM_032782.3
HCK	Probe	NM_002110.2
HLA-A	Probe	NM_002116.5
HLA-B	Probe	NM_005514.6
HLA-C	Probe	NM_002117.4
HLA-DMA	Probe	NM_006120.3
HLA-DMB	Probe	NM_002118.3
HLA-DOB	Probe	NM_002120.3
HLA-DPA1	Probe	NM_033554.2
HLA-DPB1	Probe	NM_002121.4
HLA-DQA1	Probe	NM_002122.3
HLA-DQB1	Probe	NM_002123.3
HLA-DRA	Probe	NM_019111.3
HLA-DRB3	Probe	NM_022555.3
HLA-DRB4	Probe	NM_021983.4
HLA-E	Probe	NM_005516.4
HLA-G	Probe	NM_002127.4
HMGB1	Probe	NM_002128.4
HRAS	Probe	NM_005343.2
HSD11B1	Probe	NM_181755.1
ICAM1	Probe	NM_000201.2
ICAM2	Probe	NM_000873.3
ICAM3	Probe	NM_002162.3
ICAM4	Probe	NM_001039132.1
ICOS	Probe	NM_012092.2
ICOSLG	Probe	NM_015259.4
IDO1	Probe	NM_002164.3
IFI16	Probe	NM_005531.1
IFI27	Probe	NM_005532.3
IFI35	Probe	NM_005533.3
IFIH1	Probe	NM_022168.2
IFIT1	Probe	NM_001548.3

IFIT2	Probe	NM_001547.4
IFITM1	Probe	NM_003641.3
IFITM2	Probe	NM_006435.2
IFNA1	Probe	NM_024013.1
IFNA17	Probe	NM_021268.2
IFNA2	Probe	NM_000605.3
IFNA7	Probe	NM_021057.2
IFNA8	Probe	NM_002170.3
IFNAR1	Probe	NM_000629.2
IFNAR2	Probe	NM_000874.3
IFNB1	Probe	NM_002176.2
IFNG	Probe	NM_000619.2
IFNGR1	Probe	NM_000416.1
IFNL1	Probe	NM_172140.1
IFNL2	Probe	NM_172138.1
IGF1R	Probe	NM_000875.2
IGF2R	Probe	NM_000876.1
IGLL1	Probe	NM_020070.2
IKBKB	Probe	NM_001556.1
IKBKE	Probe	NM_014002.2
IKBKG	Probe	NM_003639.2
IL10	Probe	NM_000572.2
IL10RA	Probe	NM_001558.2
IL11	Probe	NM_000641.2
IL11RA	Probe	NM_147162.1
IL12A	Probe	NM_000882.2
IL12B	Probe	NM_002187.2
IL12RB1	Probe	NM_005535.1
IL12RB2	Probe	NM_001559.2
IL13	Probe	NM_002188.2
IL13RA1	Probe	NM_001560.2
IL13RA2	Probe	NM_000640.2
IL15	Probe	NM_172174.1
IL15RA	Probe	NM_002189.2
IL16	Probe	NM_004513.4
IL17A	Probe	NM_002190.2
IL17B	Probe	NM_014443.2
IL17F	Probe	NM_052872.3
IL17RA	Probe	NM_014339.6
IL17RB	Probe	NM_018725.3
IL18	Probe	NM_001562.2
IL18R1	Probe	NM_003855.2
IL18RAP	Probe	NM_003853.2
IL19	Probe	NM_013371.3
IL1A	Probe	NM_000575.3
IL1B	Probe	NM_000576.2

IL1R1	Probe	NM_000877.2
IL1R2	Probe	NM_173343.1
IL1RAP	Probe	NM_002182.2
IL1RAPL2	Probe	NM_017416.1
IL1RL1	Probe	NM_016232.4
IL1RL2	Probe	NM_003854.2
IL1RN	Probe	NM_000577.3
IL2	Probe	NM_000586.2
IL21	Probe	NM_021803.2
IL21R	Probe	NM_021798.2
IL22	Probe	NM_020525.4
IL22RA1	Probe	NM_021258.2
IL22RA2	Probe	NM_181310.1
IL23A	Probe	NM_016584.2
IL23R	Probe	NM_144701.2
IL24	Probe	NM_181339.1
IL25	Probe	NM_022789.2
IL26	Probe	NM_018402.1
IL27	Probe	NM_145659.3
IL2RA	Probe	NM_000417.1
IL2RB	Probe	NM_000878.2
IL2RG	Probe	NM_000206.1
IL3	Probe	NM_000588.3
IL32	Probe	NM_004221.4
IL34	Probe	NM_152456.1
IL3RA	Probe	NM_002183.2
IL4	Probe	NM_000589.2
IL4R	Probe	NM_000418.2
IL5	Probe	NM_000879.2
IL5RA	Probe	NM_000564.3
IL6	Probe	NM_000600.1
IL6R	Probe	NM_000565.2
IL6ST	Probe	NM_002184.2
IL7	Probe	NM_000880.2
IL7R	Probe	NM_002185.2
IL8	Probe	NM_000584.2
IL9	Probe	NM_000590.1
ILF3	Probe	NM_001137673.1
INPP5D	Probe	NM_005541.3
IRAK1	Probe	NM_001569.3
IRAK2	Probe	NM_001570.3
IRAK4	Probe	NM_016123.1
IRF1	Probe	NM_002198.1
IRF2	Probe	NM_002199.3
IRF3	Probe	NM_001571.5
IRF4	Probe	NM_002460.1

IRF5	Probe	NM_002200.3
IRF7	Probe	NM_001572.3
IRF8	Probe	NM_002163.2
IRGM	Probe	NM_001145805.1
ISG15	Probe	NM_005101.3
ISG20	Probe	NM_002201.4
ITCH	Probe	NM_001257138.1
ITGA1	Probe	NM_181501.1
ITGA2	Probe	NM_002203.2
ITGA2B	Probe	NM_000419.3
ITGA4	Probe	NM_000885.4
ITGA5	Probe	NM_002205.2
ITGA6	Probe	NM_000210.1
ITGAE	Probe	NM_002208.4
ITGAL	Probe	NM_002209.2
ITGAM	Probe	NM_000632.3
ITGAX	Probe	NM_000887.3
ITGB1	Probe	NM_033666.2
ITGB2	Probe	NM_000211.2
ITGB3	Probe	NM_000212.2
ITGB4	Probe	NM_001005731.1
ITK	Probe	NM_005546.3
JAK1	Probe	NM_002227.1
JAK2	Probe	NM_004972.2
JAK3	Probe	NM_000215.2
JAM3	Probe	NM_032801.3
KIR_Activating_Subgroup_1	Probe	NM_001083539.1
KIR_Activating_Subgroup_2	Probe	NM_014512.1
KIR_Inhibiting_Subgroup_1	Probe	NM_014218.2
KIR_Inhibiting_Subgroup_2	Probe	NM_014511.3
KIR3DL1	Probe	NM_013289.2
KIR3DL2	Probe	NM_006737.2
KIR3DL3	Probe	NM_153443.3
KIT	Probe	NM_000222.2
KLRB1	Probe	NM_002258.2
KLRC1	Probe	NM_002259.3
KLRC2	Probe	NM_002260.3
KLRD1	Probe	NM_002262.3
KLRF1	Probe	NM_016523.1
KLRG1	Probe	NM_005810.3
KLRK1	Probe	NM_007360.3
LAG3	Probe	NM_002286.5
LAIR2	Probe	NM_002288.3
LAMP1	Probe	NM_005561.3
LAMP2	Probe	NM_001122606.1
LAMP3	Probe	NM_014398.3

LBP	Probe	NM_004139.2
LCK	Probe	NM_005356.2
LCN2	Probe	NM_005564.3
LCP1	Probe	NM_002298.4
LGALS3	Probe	NM_001177388.1
LIF	Probe	NM_002309.3
LILRA1	Probe	NM_006863.1
LILRA4	Probe	NM_012276.3
LILRA5	Probe	NM_181879.2
LILRB1	Probe	NM_001081637.1
LILRB2	Probe	NM_005874.1
LILRB3	Probe	NM_006864.2
LRP1	Probe	NM_002332.2
LRRN3	Probe	NM_001099660.1
LTA	Probe	NM_000595.2
LTB	Probe	NM_002341.1
LTBR	Probe	NM_002342.1
LTF	Probe	NM_002343.2
LTK	Probe	NM_001135685.1
LY86	Probe	NM_004271.3
LY9	Probe	NM_001033667.1
LY96	Probe	NM_015364.2
LYN	Probe	NM_002350.1
MAF	Probe	NM_005360.4
MAGEA1	Probe	NM_004988.4
MAGEA12	Probe	NM_001166386.1
MAGEA3	Probe	NM_005362.3
MAGEA4	Probe	NM_001011548.1
MAGEB2	Probe	NM_002364.4
MAGEC1	Probe	NM_005462.4
MAGEC2	Probe	NM_016249.3
MAP2K1	Probe	NM_002755.2
MAP2K2	Probe	NM_030662.2
MAP2K4	Probe	NM_003010.2
MAP3K1	Probe	NM_005921.1
MAP3K5	Probe	NM_005923.3
MAP3K7	Probe	NM_145333.1
MAP4K2	Probe	NM_004579.2
MAPK1	Probe	NM_138957.2
MAPK11	Probe	NM_002751.5
MAPK14	Probe	NM_001315.1
MAPK3	Probe	NM_001040056.1
MAPK8	Probe	NM_002750.2
MAPKAPK2	Probe	NM_004759.3
MARCO	Probe	NM_006770.3
MASP1	Probe	NM_139125.3

MASP2	Probe	NM_139208.1
MAVS	Probe	NM_020746.3
MBL2	Probe	NM_000242.2
MCAM	Probe	NM_006500.2
MEF2C	Probe	NM_002397.3
MEFV	Probe	NM_000243.2
MERTK	Probe	NM_006343.2
MFGE8	Probe	NM_001114614.1
MICA	Probe	NM_000247.1
MICB	Probe	NM_005931.3
MIF	Probe	NM_002415.1
MME	Probe	NM_000902.2
MNX1	Probe	NM_005515.3
MPPED1	Probe	NM_001044370.1
MR1	Probe	NM_001531.2
MRC1	Probe	NM_002438.2
MS4A1	Probe	NM_152866.2
MS4A2	Probe	NM_000139.3
MSR1	Probe	NM_002445.3
MST1R	Probe	NM_002447.1
MUC1	Probe	NM_001018017.1
MX1	Probe	NM_002462.2
MYD88	Probe	NM_002468.3
NCAM1	Probe	NM_000615.5
NCF4	Probe	NM_000631.4
NCR1	Probe	NM_004829.5
NEFL	Probe	NM_006158.3
NFATC1	Probe	NM_172389.1
NFATC2	Probe	NM_012340.3
NFATC3	Probe	NM_004555.2
NFATC4	Probe	NM_001136022.2
NFKB1	Probe	NM_003998.2
NFKB2	Probe	NM_002502.2
NFKBIA	Probe	NM_020529.1
NLRC5	Probe	NM_032206.4
NLRP3	Probe	NM_001079821.2
NOD1	Probe	NM_006092.1
NOD2	Probe	NM_022162.1
NOS2A	Probe	NM_153292.1
NOTCH1	Probe	NM_017617.3
NRP1	Probe	NM_003873.5
NT5E	Probe	NM_002526.2
NUP107	Probe	NM_020401.2
OAS3	Probe	NM_006187.2
OSM	Probe	NM_020530.4
PASD1	Probe	NM_173493.2

PAX5	Probe	NM_016734.1
PBK	Probe	NM_018492.2
PDCD1	Probe	NM_005018.1
PDCD1LG2	Probe	NM_025239.3
PDGFC	Probe	NM_016205.2
PDGFRB	Probe	NM_002609.3
PECAM1	Probe	NM_000442.3
PIK3CD	Probe	NM_005026.3
PIK3CG	Probe	NM_002649.2
PIN1	Probe	NM_006221.2
PLA2G1B	Probe	NM_000928.2
PLA2G6	Probe	NM_001004426.1
PLAU	Probe	NM_002658.2
PLAUR	Probe	NM_001005376.1
PMCH	Probe	NM_002674.2
PNMA1	Probe	NM_006029.4
POU2AF1	Probe	NM_006235.2
POU2F2	Probe	NM_002698.2
PPARG	Probe	NM_015869.3
PPBP	Probe	NM_002704.2
PRAME	Probe	NM_006115.3
PRF1	Probe	NM_005041.3
PRG2	Probe	NM_002728.4
PRKCD	Probe	NM_006254.3
PRKCE	Probe	NM_005400.2
PRM1	Probe	NM_002761.2
PSEN1	Probe	NM_000021.2
PSEN2	Probe	NM_000447.2
PSMB10	Probe	NM_002801.2
PSMB7	Probe	NM_002799.2
PSMB8	Probe	NM_004159.4
PSMB9	Probe	NM_002800.4
PSMD7	Probe	NM_002811.3
PTGDR2	Probe	NM_004778.1
PTGS2	Probe	NM_000963.1
PTPRC	Probe	NM_080921.3
PVR	Probe	NM_006505.3
PYCARD	Probe	NM_013258.3
RAG1	Probe	NM_000448.2
REL	Probe	NM_002908.2
RELA	Probe	NM_021975.2
RELB	Probe	NM_006509.2
REPS1	Probe	NM_001128617.2
RIPK2	Probe	NM_003821.5
ROPN1	Probe	NM_017578.2
RORA	Probe	NM_134261.2

RORC	Probe	NM_001001523.1
RPS6	Probe	NM_001010.2
RRAD	Probe	NM_004165.1
RUNX1	Probe	NM_001754.4
RUNX3	Probe	NM_004350.1
S100A12	Probe	NM_005621.1
S100A7	Probe	NM_002963.2
S100A8	Probe	NM_002964.3
S100B	Probe	NM_006272.1
SAA1	Probe	NM_199161.1
SBNO2	Probe	NM_014963.2
SELE	Probe	NM_000450.2
SELL	Probe	NR_029467.1
SELPLG	Probe	NM_001206609.1
SEMG1	Probe	NM_003007.2
SERPINB2	Probe	NM_002575.1
SERPING1	Probe	NM_000062.2
SH2B2	Probe	NM_020979.3
SH2D1A	Probe	NM_001114937.2
SH2D1B	Probe	NM_053282.4
SIGIRR	Probe	NM_021805.2
SIGLEC1	Probe	NM_023068.3
SLAMF1	Probe	NM_003037.2
SLAMF6	Probe	NM_001184714.1
SLAMF7	Probe	NM_021181.3
SLC11A1	Probe	NM_000578.2
SMAD2	Probe	NM_005901.5
SMAD3	Probe	NM_005902.3
SMPD3	Probe	NM_018667.3
SOCS1	Probe	NM_003745.1
SPA17	Probe	NM_017425.3
SPACA3	Probe	NM_173847.3
SPANXB1	Probe	NM_032461.2
SPINK5	Probe	NM_006846.3
SPN	Probe	NM_003123.3
SPO11	Probe	NM_198265.1
SPP1	Probe	NM_000582.2
SSX1	Probe	NM_005635.2
SSX4	Probe	NM_005636.3
ST6GAL1	Probe	NM_003032.2
STAT1	Probe	NM_007315.2
STAT2	Probe	NM_005419.2
STAT3	Probe	NM_139276.2
STAT4	Probe	NM_003151.2
STAT5B	Probe	NM_012448.3
STAT6	Probe	NM_003153.3

SYCP1	Probe	NM_003176.2
SYK	Probe	NM_003177.3
SYT17	Probe	NM_016524.2
TAB1	Probe	NM_153497.2
TAL1	Probe	NM_003189.2
TANK	Probe	NM_004180.2
TAP1	Probe	NM_000593.5
TAP2	Probe	NM_000544.3
TAPBP	Probe	NM_003190.4
TARP	Probe	NM_001003799.1
TBK1	Probe	NM_013254.2
TBX21	Probe	NM_013351.1
TCF7	Probe	NM_003202.2
TFE3	Probe	NM_006521.3
TFEB	Probe	NM_007162.2
TFRC	Probe	NM_003234.1
TGFB1	Probe	NM_000660.3
TGFB2	Probe	NM_003238.2
THBD	Probe	NM_000361.2
THBS1	Probe	NM_003246.2
THY1	Probe	NM_006288.2
TICAM1	Probe	NM_014261.1
TICAM2	Probe	NM_021649.4
TIGIT	Probe	NM_173799.2
TIRAP	Probe	NM_148910.2
TLR1	Probe	NM_003263.3
TLR10	Probe	NM_030956.2
TLR2	Probe	NM_003264.3
TLR3	Probe	NM_003265.2
TLR4	Probe	NM_138554.2
TLR5	Probe	NM_003268.3
TLR6	Probe	NM_006068.2
TLR7	Probe	NM_016562.3
TLR8	Probe	NM_016610.2
TLR9	Probe	NM_017442.2
TMEFF2	Probe	NM_016192.2
TNF	Probe	NM_000594.2
TNFAIP3	Probe	NM_006290.2
TNFRSF10B	Probe	NM_003842.3
TNFRSF10C	Probe	NM_003841.3
TNFRSF11A	Probe	NM_003839.2
TNFRSF11B	Probe	NM_002546.2
TNFRSF12A	Probe	NM_016639.1
TNFRSF13B	Probe	NM_012452.2
TNFRSF13C	Probe	NM_052945.3
TNFRSF14	Probe	NM_003820.2

TNFRSF17	Probe	NM_001192.2
TNFRSF18	Probe	NM_004195.2
TNFRSF1A	Probe	NM_001065.2
TNFRSF1B	Probe	NM_001066.2
TNFRSF4	Probe	NM_003327.2
TNFRSF8	Probe	NM_152942.2
TNFRSF9	Probe	NM_001561.4
TNFSF10	Probe	NM_003810.2
TNFSF11	Probe	NM_003701.2
TNFSF12	Probe	NM_003809.2
TNFSF13	Probe	NM_003808.3
TNFSF13B	Probe	NM_006573.4
TNFSF14	Probe	NM_003807.3
TNFSF15	Probe	NM_001204344.1
TNFSF18	Probe	NM_005092.2
TNFSF4	Probe	NM_003326.2
TNFSF8	Probe	NM_001244.3
TOLLIP	Probe	NM_019009.2
TP53	Probe	NM_000546.2
TPSAB1	Probe	NM_003294.3
TPTE	Probe	NM_199259.2
TRAF2	Probe	NM_021138.3
TRAF3	Probe	NM_145725.1
TRAF6	Probe	NM_145803.1
TREM1	Probe	NM_018643.3
TREM2	Probe	NM_018965.3
TTK	Probe	NM_003318.3
TXK	Probe	NM_003328.1
TXNIP	Probe	NM_006472.1
TYK2	Probe	NM_003331.3
UBC	Probe	NM_021009.3
ULBP2	Probe	NM_025217.2
USP9Y	Probe	NM_004654.3
VCAM1	Probe	NM_001078.3
VEGFA	Probe	NM_001025366.1
VEGFC	Probe	NM_005429.2
XCL2	Probe	NM_003175.3
XCR1	Probe	NM_005283.2
YTHDF2	Probe	NM_001172828.1
ZAP70	Probe	NM_001079.3
ZNF205	Probe	NM_001031686.1
ABCF1	Internal Reference	NM_001090.2
AGK	Internal Reference	NM_018238.3
ALAS1	Internal Reference	NM_000688.4
AMMECR1L	Internal Reference	NM_001199140.1
CC2D1B	Internal Reference	NM_032449.2

CNOT10	Internal Reference	NM_001256741.1
CNOT4	Internal Reference	NM_001190848.1
COG7	Internal Reference	NM_153603.3
DDX50	Internal Reference	NM_024045.1
DHX16	Internal Reference	NM_001164239.1
DNAJC14	Internal Reference	NM_032364.5
EDC3	Internal Reference	NM_001142443.1
EIF2B4	Internal Reference	NM_172195.3
ERCC3	Internal Reference	NM_000122.1
FCF1	Internal Reference	NM_015962.4
G6PD	Internal Reference	NM_000402.2
GPATCH3	Internal Reference	NM_022078.2
GUSB	Internal Reference	NM_000181.1
HDAC3	Internal Reference	NM_003883.2
HPRT1	Internal Reference	NM_000194.1
MRPS5	Internal Reference	NM_031902.3
MTMR14	Internal Reference	NM_022485.3
NOL7	Internal Reference	NM_016167.3
NUBP1	Internal Reference	NM_001278506.1
POLR2A	Internal Reference	NM_000937.2
PPIA	Internal Reference	NM_021130.2
PRPF38A	Internal Reference	NM_032864.3
SAP130	Internal Reference	NM_024545.3
SDHA	Internal Reference	NM_004168.1
SF3A3	Internal Reference	NM_006802.2
TBP	Internal Reference	NM_001172085.1
TLK2	Internal Reference	NM_006852.2
TMUB2	Internal Reference	NM_024107.2
TRIM39	Internal Reference	NM_021253.3
TUBB	Internal Reference	NM_178014.2
USP39	Internal Reference	NM_001256725.1
ZC3H14	Internal Reference	NM_001160103.1
ZKSCAN5	Internal Reference	NM_014569.3
ZNF143	Internal Reference	NM_003442.5
ZNF346	Internal Reference	NM_012279.2

Supplementary Table S1: NanoString nCounter® Human PanCancer Immune Profiling Panel. Table of genes included in the gene expression profiling panel used in this study.

GATA2 gene location	Genotype	
Exon 2	c.161C>A	p.S54X
Exon 3	c.303delC	p.A103Qfs*16
	c.341delA	p.N114Tfs*5
	c.351C>G	p.T117T
	c.416_417delCT	p.S139CfsTer45
	c.414_417delCTCT	p.S139CfsX78
	c.448G>T	p.G150*
	C599delG	p.G200VfsX18
	c.599dupG	p.S201*
Exon 4	c.981G>A	p.G327G (within N-ZF)
Intron 4-5	c.1018-10_1037del30bp	
Exon 5	c.1037_1046del	p.G346Vfs*38 (between C-ZF and N-ZF)
	c.1045T>A	p.C349S (within C-ZF)
	c. 1061C>T	p.T354M (within C-ZF)
	c.1084C>T	p.R362* (within C-ZF)
	c.1084C>T	p.R362fsx* (within C-ZF)
	c.1109G>T	p.C370F (within C-ZF)
	c.1113C>A	p.N371K (within C-ZF)
	c.1128C>G	p.Y376X (within C-ZF)
Intron 5-6	c.1143+1G>A	
Exon 6	c.1186C>T	p.R396W (within C-ZF)
	c.1187G>A	p.R396Q (within C-ZF)
	c.1215G>T	p.K405N

Supplementary Table S2: Distinct mutations found in pediatric patients with a GATA2 deficiency and their localization on the *GATA2* gene. Table of 23 distinct mutations identified in n=25 patients with GATA2 deficiency. Genotype unknown for n=5 patients. C-ZF: C-zinc finger; N-ZF: N-zinc finger.

Differentially expressed gene in GATA2-RCC versus GATA2-EB	Log2 fold change GATA2-RCC versus GATA2-EB	Std error (log2) GATA2-RCC versus GATA2-EB
BCL2	-1.200	0.294
BMI1	-1.400	0.312
CCL18	-2.140	0.232
CCRL2	1.220	0.281
CD34	-3.420	0.540
CXCR1	1.810	0.283
IFNL1	0.876	0.179
IL18RAP	2.860	0.480
LCN2	2.680	0.464
LGALS3	2.100	0.363
MEF2C	-1.760	0.430
OSM	1.230	0.250
REPS1	-1.310	0.296
TRFC	2.000	0.497
TXK	-1.240	0.278

Supplementary Table S3: Gene expression data for the 15 most differentially expressed genes in patients with a GATA2 deficiency in early and advanced disease stage in alphabetical order. GATA2-RCC: refractory cytopenia of childhood with additional *GATA2* mutation; GATA2-EB: MDS with excess blasts with additional *GATA2* mutation.

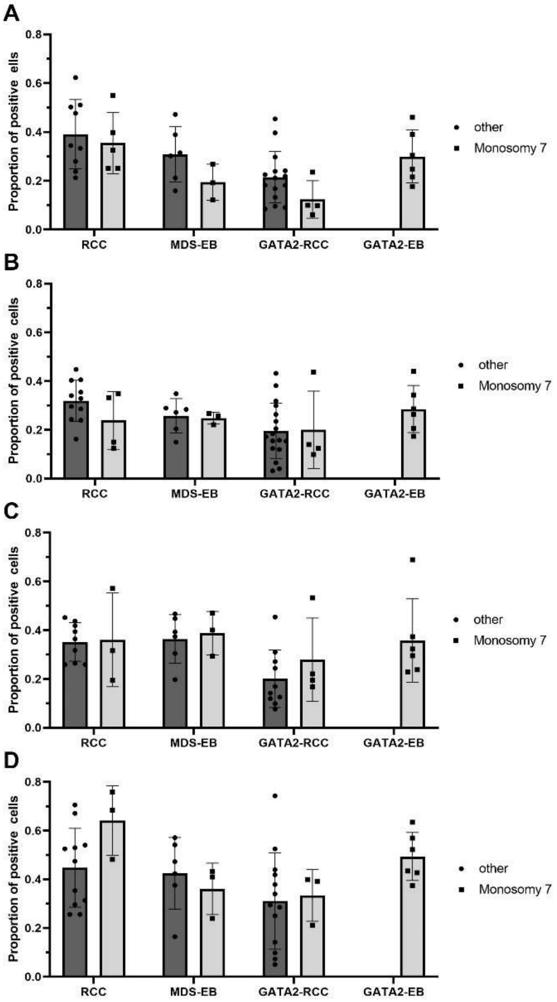

Supplementary Figure S1: Impact of the karyotype of patients on the expression of GATA2 target genes. *In situ* hybridization of GATA2 target genes *RUNX1*, *EZH2*, *IKZF1*, and *LYL1* for patients diagnosed with refractory cytopenia of childhood (RCC) or myelodysplastic syndrome with excess blasts (MDS-EB) and patients with RCC or MDS-EB and additional *GATA2* mutation (GATA2-RCC, GATA2-EB). Quantification of *RUNX1*- **(A)**, *EZH2*- **(B)**, *IKZF1*- **(C)**, and *LYL1*-positive cells **(D)** revealed no statistically significant differences for patients with monosomy 7 or other karyotypes (normal karyotype, trisomy 8 and other cytogenetic aberrations) among pediatric patients. RCC: Monosomy 7 n=5, other n=12; MDS-EB: Monosomy 7 n=3, other n=7; GATA2-RCC: Monosomy 7 n=5, other n=16; GATA2-EB: Monosomy 7 n=6, other n=0.

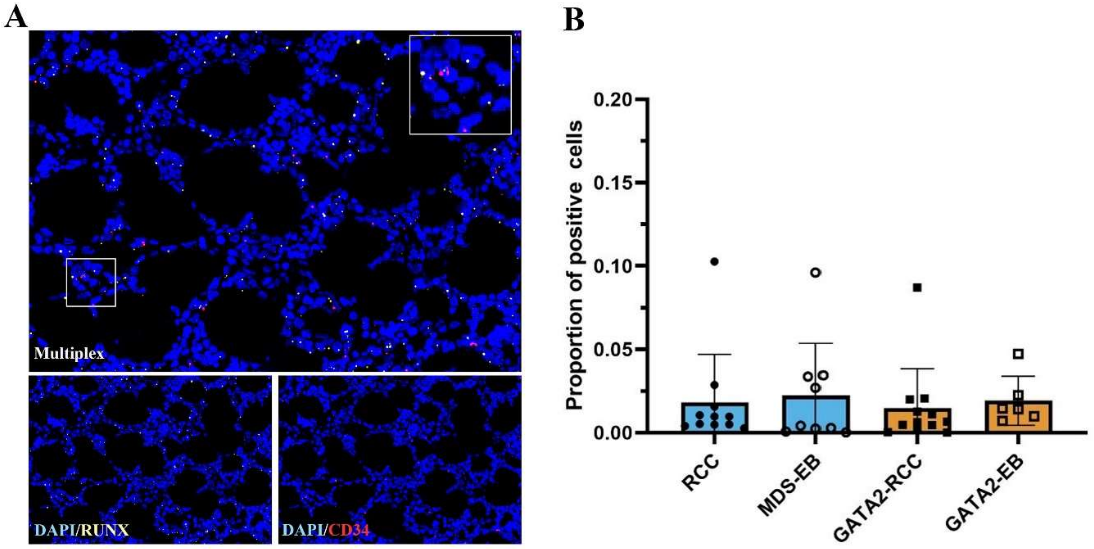

Supplementary Figure S2: Comparable expression of *RUNX1*-positive hematopoietic progenitors in all patient groups, regardless of *GATA2* mutational status and disease stage. Multiplex *in situ* hybridization **(A)** of *RUNX1* (yellow) and *CD34* (red) with DAPI counterstain (blue). Quantification of *RUNX1*-positive hematopoietic progenitors **(B)** does not vary across all four patient groups, regardless of *GATA2* mutational status or disease progression.
